# Chloride of Sodium in Visceral Obstructions

**Published:** 1853-01

**Authors:** C. W. Davis

**Affiliations:** Carlisle, Indiana


ARTICLE II.—*Chloride of Sodium in Visceral Obstructions*, by C. W. DAVIS, M. D., Carlisle, Indiana.

THE sequelæ of long and protracted cases of intermittent fever are (as it has long since been determined) visceral indurations, and in many cases an anasarcal condition of the general system. This is more apt to be the case when the disease assumes the quartan type, or as it is vulgarly termed, third day chills. . These sequelæ are, I am convinced (from the best of reasons and testimony), brought about too frequently from the injudicious use of Quinine. I state this merely as a matter of fact, not intending at this time to substantiate the conclusion from physiological evidence.

The consequences of chronic ague will, to a great degree, be found very obstinate to remedy, and frequently dangerous in their results. After our common alterative and resolvent remedies, febrifuges, &c., seem to have lost every virtue, the Chloride of Sodium will be found very efficient in arresting the disease permanently—acting as a powerful discutient—resolving the visceral enlargements—and bringing about a normal condition of the digestive viscera. I was induced to try this remedy, after the use of Mercury, Quinine, Iron, Iodine, &c., failed to do any good.

The first case was a lady of about fifty years of age, of a nervous-lymphatic habit. She had been laboring under quartan ague for some nine months, occasionally checking the paroxysms with our common febrifuges, Quinine, Iron, &c.; but finally these remedies were found inefficient, and she applied to me for remedial aid.

The diagnostic marks of this case were indicative of a vitiated and depraved condition of the circulatory, nervous, and glandular systems, connected, as is usual, with symptoms of hysteria. She complained of having no appetite, of irregularity of the bowels, and an obtuse pain in the region of the spleen and liver, (these viscera being in a state of complete induration). I prescribed various remedies, and failing to do any good, made use of the Chloride of Sodium, more from its resolvent than tonic powers.

In some nine days my patient was free from visceral enlargements, and in possession of a remarkably fine appetite. This case, as well as a number of consecutive ones, of the same cachectic habits, being restored to health by the Chloride of Sodium, bears ample testimony of the utility to this remedy in the treatment of such cases.

I take as much of the Chloride as can be dissolved in the best Cogniac Brandy, and direct the patient to take a tea-spoonful three times per day before eating, using the saline bath two or three times during the week.

